# The family medicine accelerated track at Texas Tech University Health Sciences Center: results from a 10-year program to bend the primary care curve

**DOI:** 10.1080/10872981.2025.2457783

**Published:** 2025-01-24

**Authors:** Betsy Goebel Jones, Ronald C. Cook, Felix Morales, Keeley Hobart, Steven L. Berk

**Affiliations:** a Department of Medical Education, Family Medicine Accelerated Track, Texas Tech University Health Sciences Center, Lubbock, TX, USA; b FMAT Program Steering Committee, School of Medicine, Texas Tech University Health Sciences Center, Lubbock, TX, USA; c Department of Family & Community Medicine, Family Medicine Accelerated Track, Texas Tech University Health Sciences Center, Lubbock, TX, USA; d School of Medicine, Texas Tech University Health Sciences Center, Lubbock, TX, USA

**Keywords:** Accelerated training, 3-year MD, primary care workforce, medical school cost, family medicine

## Abstract

**Background:**

Texas is one of the states with the lowest access to usual sources of primary care; most critically, family medicine (FM) has been projected to have the greatest physician shortage increase between 2018 and 2032. Texas Tech University Health Sciences Center (TTUHSC) School of Medicine developed the Family Medicine Accelerated Track (FMAT), a 3-year curriculum that culminates in the MD degree and links medical students to FM residency programs at TTUHSC campuses in Lubbock, Amarillo or the Permian Basin. This article reflects on 10 years of experience with the program, and particularly its impact on the primary care physician workforce in Texas.

**Curriculum Design:**

TTUHSC medical students in the traditional curriculum complete the Phase 1 pre-clinical curriculum in Lubbock and are distributed for Phases 2 and 3 (MS3/MS4 years) among campuses in Lubbock, Amarillo and the Permian Basin. Similarly, FMAT students complete Phase 1 in Lubbock. For Phase 2 clinical clerkships, their curriculum is delivered on the campus (which may include Lubbock) where they will typically complete 3 years of FM residency training.

**Program Outcomes:**

In the 2 years prior to the graduation of the first FMAT class, just over 11% of the graduating class matched into FM. In the decade since, the numbers have varied from year to year (often as high as 17–19% of the class matching into FM) but have always exceeded the pre-FMAT numbers. For the classes 2013 through 2023, 115 students began FMAT training; 90 of them (78%) graduated in 3 years with the MD degree and began FM residency training. Of those 90, 56 have now graduated from residency and taken positions in the primary care physician workforce. Of that group, 86% are practicing in Texas, 64% are in West Texas, and 69% are in rural or underserved communities.

## Defining the health care workforce future

Medical education plays the ‘long game’ – the work that medical educators do now in recruiting, educating, and mentoring young learners bears fruit only years later, when those learners finish medical school, residency, fellowship and begin their own careers in patient care, industry, or their own teaching of another generation. Thus, planning for the long-term needs in health care, medical education, and workforce also demands a fair amount of clairvoyance about the future. Management consultant Peter Drucker is widely quoted as having said that ‘trying to predict the future is like trying to drive down a country road at night with no lights while looking out the back window.’ Available data, however, do provide some reliable indicators for the road visible from the front window. A recent report from the Robert Graham Center reminds us that ‘adults with a primary care physician have higher rates of preventive care, have improved communication with their care team, and receive more attention to social needs.’ But the report points out that the overall percentage of U.S. patients reporting a usual source of care declined from 84% in 2000 to 74% in 2019 and that access to a usual source of primary care varies widely among states [1].

Texas is one of the states with the lowest access to ‘usual sources of care,’ defined by Agency for Healthcare Research and Quality (AHRQ) as ‘the particular medical professional, doctor’s office, clinic, health center, or other place where a person would usually go if sick or in need of advice about his or her health’ [2]: The Health Professions Resource Center at the Texas Department of State Health Services (DSHS), in its supply and demand projections for 2018 through 2032, finds that the shortage of all physicians in Texas is projected to increase from 6,218 full-time equivalents (FTEs) in 2018 to 10,330 FTEs in 2032. Moreover, among the 35 physician specialties studied, family medicine (FM) was projected to have the greatest shortage increase over that period, from 1,034 FTEs in 2018 to 2,495 in 2032 [3].

Complicating this widening gap in the FM physician workforce is the concurrent desire to ‘bend the cost curve’ in health care, specifically by increasing the number of primary care physicians in general and FM physicians in particular, given the need to provide preventive care, support continuity, manage chronic disease, and reduce emergency room visits [4,5].

In the face of these insistent calls for an expanded primary care workforce and a re-balanced physician workforce in favor of primary care [6–11], Texas Tech University Health Sciences Center (TTUHSC) School of Medicine developed the Family Medicine Accelerated Track (FMAT), a 3-year curriculum that culminates in the MD degree and links medical students to family medicine residency programs at Texas Tech University Health Sciences Center (TTUHSC) campuses in Lubbock, Amarillo or the Permian Basin (Odessa and Midland) [12,13].

The purpose of FMAT is to prepare primary care physicians more efficiently and with less cost and associated debt to the student. To meet this purpose, FMAT focuses on family medicine (FM) specifically, rather than primary care more broadly: Nationally, more than 90% of FM residency graduates remain in primary care [14], and almost 40% do so in communities that are otherwise medically underserved. In contrast, only about 20% of Internal Medicine graduates [15] and 40% of Pediatrics graduates [16] choose primary care careers. The TTUHSC FM residency programs, in particular, have been especially successful in training primary care physicians for West Texas, a region that remains medically underserved. Of the 108 counties in TTUHSC’s catchment area, 98 are classified as rural and 25 are among the 35 that lack even one active physician [17].

The first class of FMAT students graduated from medical school a decade ago – in 2013—and from FM residency in 2016. As of 2023, 90 students have graduated from the TTUHSC School of Medicine via the FMAT program, and 56 have completed residency. Thus, we take the opportunity in this article to reflect on our 10 years of experience with the program, and particularly its impact on the primary care physician workforce in Texas.

## Accelerated training as a pathway to family medicine

Despite steady growth in the number of FM positions offered in the National Residency Matching Program (NRMP) – rising from 2,708 in 2011 to 5,107 in 2023—the percentage of FM positions relative to other specialties rose only slightly, from 11.7% to 13.6% of all positions offered in the Match. The overall fill rate in FM was 88.7%, of which 29.4% were US seniors from MD programs (whereas 29.6% were DO seniors and 26.5% were international graduates [18]).

Several theories have been proposed to explain the relatively low number of U.S. seniors choosing FM. A common explanation is financial, related both to student debt loads and to the disparity in compensation between primary care and subspecialty physicians. Recent scholarship has looked at the role of student debt on career choice, concluding that students are keenly aware of potential earning capacity among medical specialties and that their own debt ultimately drives their career choice – especially away from primary care [19–22]. Among the recommendations made by Petterson et al. to bolster a primary care pipeline is to provide medical school student debt relief [23]. The FMAT program takes on this challenge directly; FMAT students receive 1 year of scholarship support during medical school, and they begin to earn a residency salary 1 year earlier, effectively halving the cost of tuition and fees.

In addition to debt relief, TTUHSC’s decision to develop the FMAT program was driven by recognition of the several benefits to accelerated training:

Accelerated programs shorten the time and narrow the gap between medical school and practice. Surveys of our own students tell us that they are most often drawn to FMAT because it provides an earlier career start, which is especially appealing to older students who come to medicine as a second career and often have spouses and children [22].

With full institutional and leadership support, accelerated training programs enjoy enhanced and visible status within the institution, which accrues to all stakeholders – departments of FM, faculty and clinical systems that provide training, and most especially to the students selected for the programs. As a result, an accelerated training program that targets FM has the effect of increasing the status of FM (and probably other primary care fields as well) across the institution.

Accelerated training functions within tradition-bound medical education as a *disruptive innovation*, a term used by Christensen and Hwang to describe a process that creates change in established systems via products and systems that are simpler and more affordable [24]—an obvious example is a 3-year MD degree whose owner has less debt to repay.

## Description of the FMAT program

### FMAT curriculum

TTUHSC medical students in the traditional curriculum (currently 180 in each class) complete the Phase 1 pre-clinical curriculum in Lubbock and are distributed for Phases 2 and 3 (MS3/MS4 years) among two campuses in Lubbock (at University Medical Center and Covenant Health System campuses, about 57% of the class), Amarillo (30%) and the Permian Basin (13%). Similarly, FMAT students complete Phase 1 in Lubbock. For Phase 2 clinical clerkships, their curriculum is delivered on the campus (which may include Lubbock) where they will typically complete 3 years of FM residency training.

The FMAT curriculum, which was approved by the Liaison Committee for Medical Education (LCME) in 2010, is covered in an equivalent of 141 weeks, compared to the 157 weeks of the 4-year program. Specially developed FMAT courses include an 8-week systems-based experience in the summer between students’ first and second years of medical school, team-taught by family medicine and basic sciences faculty; a 7-month longitudinal family medicine clerkship that students complete during their second year, concurrent with MS2 pre-clinical courses; and a 16-week capstone experience from March through June of their MS3 year that includes hospital-based FM sub-internship and outpatient clinical experiences, ICU rotations, Step 2 study, and a transition-to-residency experience that is required of MS4 students in the traditional curriculum.

### FMAT student selection

The TTUHSC School of Medicine fills FMAT positions both during the pre-matriculation process and in the fall semester from the sitting MS1 class. Students are selected based on academic performance, interest in FM and an interview with the FMAT committee. Factors that affect class size (typically 8–20 students) are available residency slots, faculty capacity, and student interest.

### Transition from FMAT to 4-year curriculum

Students may choose to return to the traditional 4-year curriculum at any time prior to January of their MS3 year, when final residency numbers are due to the NRMP. They may do so for any number of reasons, but typically because they have decided not to pursue residency in FM. They are also given the option of going through the Match, rather than using the ‘all-in exception’ [25] (described below) to transition to a TTUHSC-FM residency program.

In addition, because the accelerated nature of the program does not allow time for remediation or delays due to personal or academic reasons, students may be counseled by the School of Medicine to return to the 4-year curriculum. For example, students who do not pass the USMLE Step 1 exam or who delay taking that exam based on predictive exam scores require additional curricular time that the FMAT program cannot accommodate.

### Transition to residency

FMAT students register with NRMP and ERAS during the fall, but this program has also qualified for the ‘Family Medicine Accelerated Programs’ exception to the NRMP’s All-In policy, which allows early placement in participating FM programs by reducing the number of residency positions in the Match; all but five graduating FMAT students have taken advantage of this exception. As noted previously, students have the option of participating in the open Match, with the recognition that without completing all of their Phase 2 clerkships or a score on the USMLE score (both of which come later in the spring), they do so at a disadvantage compared to their peers trained in 4-year curricula.

### FMAT program outcomes

In addition to providing an educational experience for our FMAT students that is comparable to the traditional 4-year curriculum, our intended outcomes for the program are:

- To increase the number of students from TTUSOM choosing residencies in FM and ultimately building careers in family medicine – ideally in those areas most in need of physicians – through both the FMAT program’s unique pathway and an increased status and visibility for primary care throughout the institution.
- To take advantage of the implementation of an accelerated training model within an established medical school to catalyze an environment and create an opening for educational innovation and new methodologies.
- To place program graduates into areas most in need of family physicians, including in rural and underserved areas of Texas.

## Meeting FMAT program outcomes

### Program recruitment and retention

FMAT training began for the first class of students in June 2011; those first eight students graduated in May 2013 began residency the following July, and completed their third and final year of FM residency in June 2015. As of fall 2023, 90 students have graduated from the program and begun FM residency (classes of 2013–2023); 40 additional students are currently in training as medical students (classes of 2024, 2025, and 2026), and recruitment is ongoing for the class of 2027. Across the 10 years in which we have had graduates, TTUHSC successfully took advantage of the FM accelerated track exception, and thus far has placed 85 FMAT graduates in our three participating TTUHSC programs, in Amarillo (22 FMAT-trained residents), Lubbock (54 residents), and the Permian Basin (9 residents). Five FMAT graduates participated in the open Match and successfully matched in five different FM programs in Texas. Table 1 lists data for the FMAT classes of 2013–2023.

**Table 1.** FMAT program recruitment, retention, and transition to residency, classes 2013–2023.

FMAT Graduation Year	Number Beginning FMAT1 Training	Number Counseled to Return to 4-Year Program	Number Choosing to Return to 4-Year Program	Number FMAT Grads Residency Training/Location
2013	9	1	0	8 *TTUHSC-Amarillo: 3* *TTUHSC-Lubbock: 5*
2014	7	1	1	5 *TTUHSC-Lubbock: 4* *Match: 1*
2015	8	1	0	7 *TTUHSC-Amarillo: 2* *TTUHSC-Lubbock: 5*
2016	15	1	3	11 *TTUHSC-Amarillo: 3* *TTUHSC-Lubbock: 7* *TTUHSC-Permian: 1*
2017	9	0	1	8 *TTUHSC-Amarillo: 1* *TTUHSC-Lubbock: 5* *TTUHSC-Permian: 1* *Match: 1*
2018	9	2	1	6 *TTUHSC-Lubbock: 6*
2019	7	1	0	6 *TTUHSC-Amarillo: 2* *TTUHSC-Lubbock: 3* *TTUHSC Permian: 1*
2020	9	1	0	8 *TTUHSC-Amarillo: 4* *TTUHSC-Lubbock: 4*
2021	12	0	4	8 *TTUHSC-Lubbock: 7* *TTUHSC-Lubbock: 1*
2022	19	1	2	16 *TTUHSC-Amarillo: 4* *TTUHSC-Lubbock: 6* *TTUHSC Permian: 4* *Match: 2*
2023	11	4	0	7 *TTUHSC-Amarillo: 3* *TTUHSC-Lubbock: 2* *TTUHSC Permian: 1* *Match: 1*
Totals, as of Fall. 2023	115	13	12	90 *TTUHSC-Amarillo: 22* *TTUHSC-Lubbock: 54* *TTUHSC Permian: 9* *Match: 5*

FMAT students’ performance on USMLE Step exams compares favorably with that of TTUHSC students as a whole. For the FMAT classes of 2013 through 2022 (all classes that took a scored Step 1), FMAT students averaged 221 on Step 1 (SD 15.0, median score 220.5). They averaged 233.5 on Step 2CK (SD 13.0, median score 236). Overall, TTUHSC students in those same classes averaged 227.8 on Step 1 and 240.4 on Step 2CK.

FMAT program retention has been high. As of Fall 2023, of the 115 students who have begun training in the FMAT program for the classes of 2013–2023, 90 graduated with the MD degree (78.2%). Twelve students (10.4%) chose to return to the 4-year curriculum – most to pursue residency training other than FM. In addition, 13 students (11.3%) were transitioned to the 4-year curriculum by the School of Medicine in order to provide additional time for academic preparation. Significantly, interest in FM remains high among students who were counseled to return to the 4-year curriculum: of those 13, 6 graduated in 4 years and all ultimately matched into FM residency. One student took a personal leave and graduated in 5 years with a match into Internal Medicine. Four of the 13 chose to leave medical school altogether. Two are on track to graduate with the traditional class of 2024, and both are planning to apply specifically to FM programs.

### Increasing numbers of TTUHSC students choosing FM

**Figure 1.**
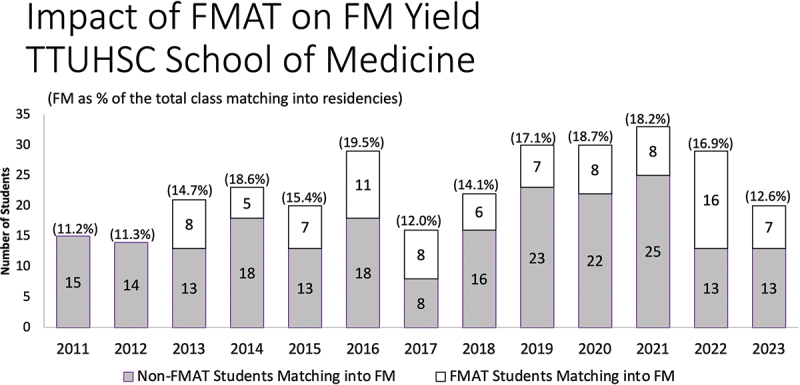
Impact of FMAT on Family Medicine Residency Match at TTUHSC.

In the NRMP Matches for 2011 and 2012, the 2 years prior to our first FMAT graduating class, 15 and 14 TTUHSC School of Medicine students, respectively, matched to FM residencies. In the 11 Matches since (2013–2023), those numbers have varied from year to year, but in all years, the ‘FM yield’ exceeds the number and percentage compared to the matches prior to 2013 (Figure 1). While we would expect a bump in numbers for the first class – which included both 4-year and 3-year program graduates – the numbers have remained strong. Thus, we do not see that the 3-year program has become the dominant route for FM residency at TTUHSC but has allowed a parallel pathway for mature students able to make early decisions about residency and specialty and receive incentives, mentoring, and support for doing so. Moreover, in an increasingly competitive Match environment, FMAT students rate highly the importance of the TTUHSC medical school-to-residency program link as a motivator to participate in the program.

While the percentage of TTUHSC School of Medicine students matching with FM in 2011 was 11.2%—close to the national average of 11.4%—the TTUHSC percentage of students matching into FM has reached as high as 19.5%. The 2023 class fell to 12.6% matching into FM overall; this class was likely the most impacted by Covid disruptions in their training. The FMAT class of 2023, in fact, lost four of the students who began FMAT training in 2021-two were counseled to return to the traditional curriculum due to Step 1 delays, and two chose to leave medical school altogether to pursue other career pathways. Certainly, the factors influencing students’ residency choices are complex and involve national trends related to reimbursement, more intense competition for positions, and the vagaries of medical student interests, and can be considered limitations to drawing conclusions about student numbers. But the strong visibility of the FMAT program in particular and family medicine in general across our institution cannot help but affect those numbers as well. Indeed, our own evaluation has revealed that the draw of an earlier beginning to a medical career is the most powerful factor in our students’ decision to apply for the FMAT program with the effect of recruiting students who ‘might not otherwise have considered family medicine’ as a specialty choice [26].

### FMAT graduate performance in residency

Before launching the program, a reasonable question posed to program leadership was whether students trained in only 3 years would transition to residency with adequate training, competence, or confidence. Our experience, as well as that of other accelerated programs, is that 3-year graduates enter residency fully ready to assume resident roles [27–30]. FMAT-trained residents have a 100% pass rate on the FM specialty board exam, and nine of them (10%) have served as their programs’ chief residents. Using the milestones developed to assess residents’ performance, FMAT graduates have been assessed as performing at or above performance levels of their peers from 4-year programs. While we anticipated some adjustments, as FMAT graduates became residents after 3 years, those adjustments have been minimal. Indeed, those students’ familiarity with clinical and hospital facilities, their ongoing relationships with faculty and other residents, and their commitment to become family physicians of at least 3 years’ standing has served them well in making an early transition from student to resident.

### FMAT as an innovation in medical education

The TTUHSC School of Medicine has clearly developed a national reputation for innovation in medical education to address physician shortages and specifically for accelerated training. In the program’s early days of development, TTUHSC Dean Steven Berk MD was interviewed by the New York Times [31], Washington Post [32], and US News and World Report [33], among others, because the FMAT program was then an exemplar for dramatic action and positive impact on the advancement of physician training. Since 2014, with the development of the Consortium for Accelerated Medical Pathway Programs (CAMPP) [28], spearheaded by the NYU Grossman School of Medicine, TTUHSC is part of a national organization likewise engaged in implementing and assessing the benefits of accelerated training as a way to reduce student debt and provide more direct pathways to increase the physician workforce more quickly across the U.S. The CAMPP consortium shares experiences and best practices in accelerated MD training and is involved in a multi-institution analysis of key data related to shared program goals, including readiness for residency, levels of burnout, and student debt [27,28]. As a pathway to a medical degree, accelerated programs again reached the notice of the US News in 2022, with a dedicated article on schools within the CAMPP consortium, including TTUHSC [34].

### Impact of the FMAT program on the primary care physician workforce

It is only now, after the FMAT program has been in place for a decade, and after multiple classes have completed their training and moved into practice and professional roles, can we observe its impact on the physician workforce. Indeed, this effort to ‘bend the curve’ toward primary care is in many ways the crowning achievement as we review graduates’ roles and practice sites. As of Fall 2023, 56 of our 90 graduates have also completed family medicine residency and launched post-residency careers. Key details about post-residency practice sites include:

- 48 (86%) of residency graduates are practicing in Texas; others are in Kansas, Louisiana, Illinois, Idaho, and Colorado.
- 33 (69%) of residency graduates took positions in rural or underserved communities (most in health professions shortage areas or HPSAs); all of these are in Texas
- 36 (64%) took positions in West Texas, specifically including the 108-county catchment area of TTUHSC
- 7 (12.5%) are in academic positions, all at TTUHSC; this includes three graduates who originally took other positions but were recruited to return to the Lubbock FM department.
- 4 (7%) completed post-residency primary care fellowships at TTUHSC, 2 in sports medicine and one each in geriatrics and hospice/palliative care medicine

A limitation of the analysis of practice site data is that, like many young physicians who have built successful practices, FMAT graduates are often recruited to new opportunities after having completed initial contractual obligations. Findings presented here represent our best data as of fall, 2023.

It is impossible to determine whether the FMAT program, alone, has increased the primary care workforce, including in rural and underserved areas of Texas beyond what might have occurred in its absence. We do, however, point back to the Texas DSHS report that predicted growing primary care physician shortages through 2032 and that report also found that Texas DSHS Region 1, which includes the Panhandle areas of Amarillo and Lubbock, was not currently a regional identified as having a ‘critical shortage’ of family medicine in 2021 [3].

## Challenges and lessons learned

### FMAT program challenges and pleasant surprises

The challenges we have experienced thus far have been few but real. Befitting a program with the word ‘accelerated’ in its title, the most common and demanding challenges are related to time – FMAT’s fast pace allows no time for remediation of courses or exams, delays due to illness or other personal issues, or experiences that otherwise enhance medical school, such as senior electives and summer global health activities. Similarly, the truncated schedule, especially in students’ MS3 year, when residency positions are set and a number of exams are taken, allows little time for faculty and administrators to identify students at academic risk for not passing those exams. Additional challenges for TTUHSC have been related to determining the best balance between FMAT and other graduates within residency programs and how best to deploy the FM positions available across all of our campuses. Nonetheless, students are willing to make the trade-offs, and each year has brought additional data to help refine schedules, policies and decision-making.

To this list of challenges, it is important to address the costs of the program that accrue to the School of Medicine and to the Health Sciences Center. Direct administrative costs include about 1.0 FTE for a program coordinator as well as funding for expenses that include study materials, student and faculty travel, meeting and graduation reception events, recruitment items, etc.; much of this cost is set and does not scale with larger or smaller class sizes. Additional costs can be calculated on a per-student basis:

- During students’ longitudinal FM clerkship during their second year, FMAT students receive a full scholarship covering tuition and fees. As of fall, 2023, this scholarship’s cost is $20,779 per student for the full academic year. Scholarship sources do include an endowment established specifically to fund FMAT scholarships, as well as other funds raised for FMAT student support.
- Also during that MS2 year, students see patients in the FM outpatient clinic, specifically during mornings regularly scheduled for faculty clinics. The full patient load for a morning faculty clinic is 10 patients; when FMAT students are included, the patient load falls to 6. Thus, we have calculated that the approximate per-student cost to FM clinic productivity is $14,400 per academic year.
- Just as the biggest benefit to students in accelerated programs is in not having a fourth year, the biggest cost to the institution is in the loss of both tuition and state-generated formula funding for that year. As noted above, the tuition that the institution forgoes is currently $20,779 per student per year. The lost amount of formula funding to the School of Medicine (which is calculated based on a formula set by the Texas legislature) is about $40,000 per student.

Over the program’s first decade, we were funded by three grants from the Health Resources Services Administration (HRSA) that helped support some program direct costs and faculty time, particularly valuable during the program’s start-up phases. As to how this per-student cost of around $100,000 in direct and opportunity costs is justified, we point to several benefits: lower student debt, strong residents entering TTUHSC FM programs, recognition of our school for its innovation and its emphasis on building the primary care workforce. Most of all, we consider the FMAT program to be an institutional priority and thus deserving of its associated costs.

What has not been challenging – despite our early concerns about opt-out or burn-out – is encouraging student interest in the program or retaining them once enrolled. As noted above, factors that lead to students’ residency selection are varied and span well beyond one medical school, but pleasant surprises have included continued high interest in FM (even among students who leave FMAT), increasing interest in FM from non-FMAT students (which we hypothesize is related to FM’s higher status and visibility within the School of Medicine), and the ability of our FM programs to recruit top candidates from the non-FMAT applicant pool. Nor have we encountered resistance from the TTUHSC faculty, including those outside the FM department. Most satisfying has been the level of performance that FMAT graduates have demonstrated as they transition to residency. They are confident, familiar with inpatient and outpatient settings, and cognizant of their roles as ambassadors for the program.

### Conclusion and the road ahead

TTUHSC’s early adoption of an accelerated pathway has opened a new road for the institution as a leader in curricular innovation that will be increasingly competency-based [28]. The road ahead for medical education is likely to be linked less to time than to demonstrating proficiency in communicating with and caring for patients, and FMAT is one vehicle on that road. Indeed, we do not argue that FMAT or any accelerated program is for every student, or even for most students destined to FM or primary care careers; rather, it is one pathway for mature and capable students who clearly see their futures as family physicians and are ready to take their place on a road, even with its bends and curves, to increase Texas’ primary care physician workforce.

## Additional information

Dr Steven Berk was Dean of the TTUHSC School of Medicine from 2006 until his death in May 2023. Dr Berk was a tireless visionary on behalf of health care in West Texas, and it was his push that launched and supported the Family Medicine Accelerated Track. He wrote and spoke often about the program, and this article includes Dr Berk’s perspectives and words on its behalf.
